# Harnessing 3D cell models and high-resolution imaging to unveil the mechanisms of nanoparticle-mediated drug delivery

**DOI:** 10.3389/fbioe.2025.1606573

**Published:** 2025-07-07

**Authors:** Alannah S. Chalkley, Maëva T. Lopez, Margaritha M. Mysior, Madeleen C. Brink, Suainibhe Kelly, Jeremy C. Simpson

**Affiliations:** Cell Screening Laboratory, UCD School of Biology and Environmental Science, University College Dublin, Dublin, Ireland

**Keywords:** nanoparticles, drug delivery, spheroids, organoids, 3D cell models, fluorescence microscopy, high-content analysis

## Abstract

Nanoparticles and nanosized materials offer huge potential in the field of drug delivery. One key aspect that dictates their successful development is the need to understand how they interact with cells at both the macro and molecular level. Delineating such interactions is vital if nanomaterials are to be targeted not only to particular organs and tissues, but also to individual cell types and ultimately specific subcellular locations. In this regard, the development of appropriate *in vitro* cell models is an essential prerequisite before animal and human trials. In recent years, as the methodology for their growth has been refined, there has been a huge expansion in the use of pre-clinical 3D cell culture models, particularly spheroids and organoids. These models are attractive because they can be combined with high-resolution fluorescence imaging to provide real-time information on how nanomaterials interact with cells. Confocal fluorescence microscopy and its associated modalities, along with high-content screening and analysis, are powerful techniques that allow researchers the possibility of extracting spatial and temporal information at multiple levels from cells and entire 3D assemblies. In this review, we summarise the state of this field, paying particular emphasis to how imaging of such models is now beginning to provide rich quantitative data about nanomaterial entry and trafficking in cells growing in 3D. We also offer a perspective on the challenges faced by such approaches, and the important questions that the drug delivery field still needs to address.

## 1 Introduction

Nanoparticles (NPs) ranging in size from 1 to 100 nm have gained traction over the years for their potential application as therapeutic delivery agents. When carrying a payload, they fall under the umbrella term of nanomedicine and hold great potential in the field of personalised medicine due to their specificity (Elumalai et al., 2024). For this potential to be fully realised, NPs require improvement in targeted delivery and enhanced drug release efficiency with reduced toxicity. These improvements ultimately seek to limit harmful side effects experienced by patients. Nanomaterials offer excellent therapeutic potential as they can be engineered to carry diverse payloads such as chemotherapeutic drugs, small molecule inhibitors and monoclonal antibodies (Xu et al., 2023). To date, over 80 NPs comprising lipids, polymers, and inorganic materials have gained European Medicines Agency and Food and Drug Administration approval for clinical use, with applications as vaccine adjuvants and treatments for cancer and inflammation (Halwani, 2022). Despite this success, the use of NPs in a clinical setting is still a cause for concern, with many unanswered questions surrounding their mechanisms of uptake, trafficking and penetration into tumours (Munir, 2022). Understanding the precise molecular effects that NPs exert on cells and the wider tissue environment creates a bottleneck for more NP-based therapies to transition into the clinical setting. Quantitative and data-rich fluorescence microscopy provides spatial information in two-dimensional (2D) and three-dimensional (3D) models, elucidates NP localisation, and can explore the effects of NPs on specific organelles and cellular pathways.

Drug development has long relied on the use of *in vitro* cell culture approaches. For several decades, this has involved the use of traditional monolayer (2D) cell culture of established cell lines, which have been widely used and have paved the way for therapeutic advances and numerous drug approvals. Given that NP-based therapeutics are composed of a wide variety of material types, it is highly likely that the effects they exert on cells will be varied (Halwani, 2022). As such, nanomedicines require extensive characterisation, assessing their targeting, uptake and penetration, mechanism of action, and general biocompatibility (Elumalai et al., 2024). While these features can be partially investigated using *in vitro* monolayer-based mammalian cell models, 3D models are increasingly providing crucial spatial information that is heavily limited within 2D models. The use of 3D models introduces effective cell-cell interactions, increases cell type complexity, and begins to create microenvironments similar to those seen in tumours (Elumalai et al., 2024; Zhou et al., 2024).

In this review, we describe how 3D cell culture systems provide the opportunity to gather robust, quantitative data similar to that obtained from 2D cell culture systems, and how 3D cell models can close the knowledge gap between information gathered from monolayer cell models and animal studies. In comparison to monolayer systems, 3D cell models are morphologically of greater relevance to the cell types and tissues that would be encountered *in vivo* by nanomedicines. Furthermore, given the range of cancer cells now available to laboratories, they present a particularly exciting opportunity for testing nanomedicines against specific cancer types. Ultimately, it is expected that 3D cell models will make it possible to obtain a clearer understanding of NP function and potential toxicity *in vitro* before moving into animal models, thereby mitigating serious, unforeseen responses *in vivo*.

This review outlines the current state of the art in the production and application of 3D cell models, highlighting their contribution to understanding NP interactions with biological systems. Additionally, we summarise the range of studies that have employed quantitative fluorescence imaging technologies to advance our understanding of how NPs penetrate 3D cell models and effect broader phenotypes on cells. These techniques not only facilitate visualisation and measurement of gross changes in 3D cell assemblies upon NP exposure, but also provide information at single-cell and subcellular resolution. Such knowledge is expected to provide the necessary spatial and temporal insights that will be crucial for enhancing our understanding of the mechanistic details of NP-cell interactions. Importantly, this refined understanding has the potential to revolutionise the field of drug delivery, enabling more precise targeting and improved therapeutic efficacy to specific cellular environments with unprecedented accuracy.

## 2 3D cell culture methods

3D cell culture approaches are increasingly being utilised in drug discovery and delivery studies, cancer biology, and fundamental research (Jensen and Teng, 2020). While conventional 2D monolayer-based studies have been instrumental in improving our understanding of cellular processes, they lack several key features of *in vivo* tissue architecture such as cell-cell contacts in all dimensions. As we test potential nanomedicines, the *in vitro* pre-clinical models need to reliably predict their performance and therapeutic delivery in human tissues (Ruedinger et al., 2015). This is particularly relevant as reports have emerged suggesting that 3D cell models can display different gene expression and drug resistance patterns compared to monolayers (Ruedinger et al., 2015).

Although there are several types of 3D cell models now being used, by far the two most common formats are spheroids and organoids. Spheroids are the simplest form of 3D cell model and are described as self-assembled aggregates of cells that can be derived from a multitude of cell types (Edmondson et al., 2014). Spheroids maintain cell-cell and cell-extracellular matrix (ECM) interactions and their complexity can be increased through the co-culture of different cell types (Roy et al., 2023). Larger spheroids (>500 µm diameter) can mimic the oxygen and nutrient gradients found in solid tumours and show differing cell behaviour between the outer and inner layers (Nunes et al., 2019). Organoids, on the other hand, are 3D cell models that are generated from stem cells, and more closely mirror the normal organ or tissue physiology found *in vivo* (Kim et al., 2020; Rathore et al., 2019). Different classes of stem cells, including pluripotent stem cells, induced pluripotent stem cells and organ-specific adult stem cells can be used to produce organoids (Yin et al., 2016). In addition to spheroids and organoids, an emerging 3D cell structure being used is the patient-derived explant, which is a tumour that is extracted from a patient and then grown *in vitro* (Powley et al., 2020). Their wider use potentially offers exciting possibilities for personalised therapeutic regimes. However, as these models are challenging to image, and have not yet been employed in any significant way in NP studies, they will not be considered further in this review.

**FIGURE 1:**
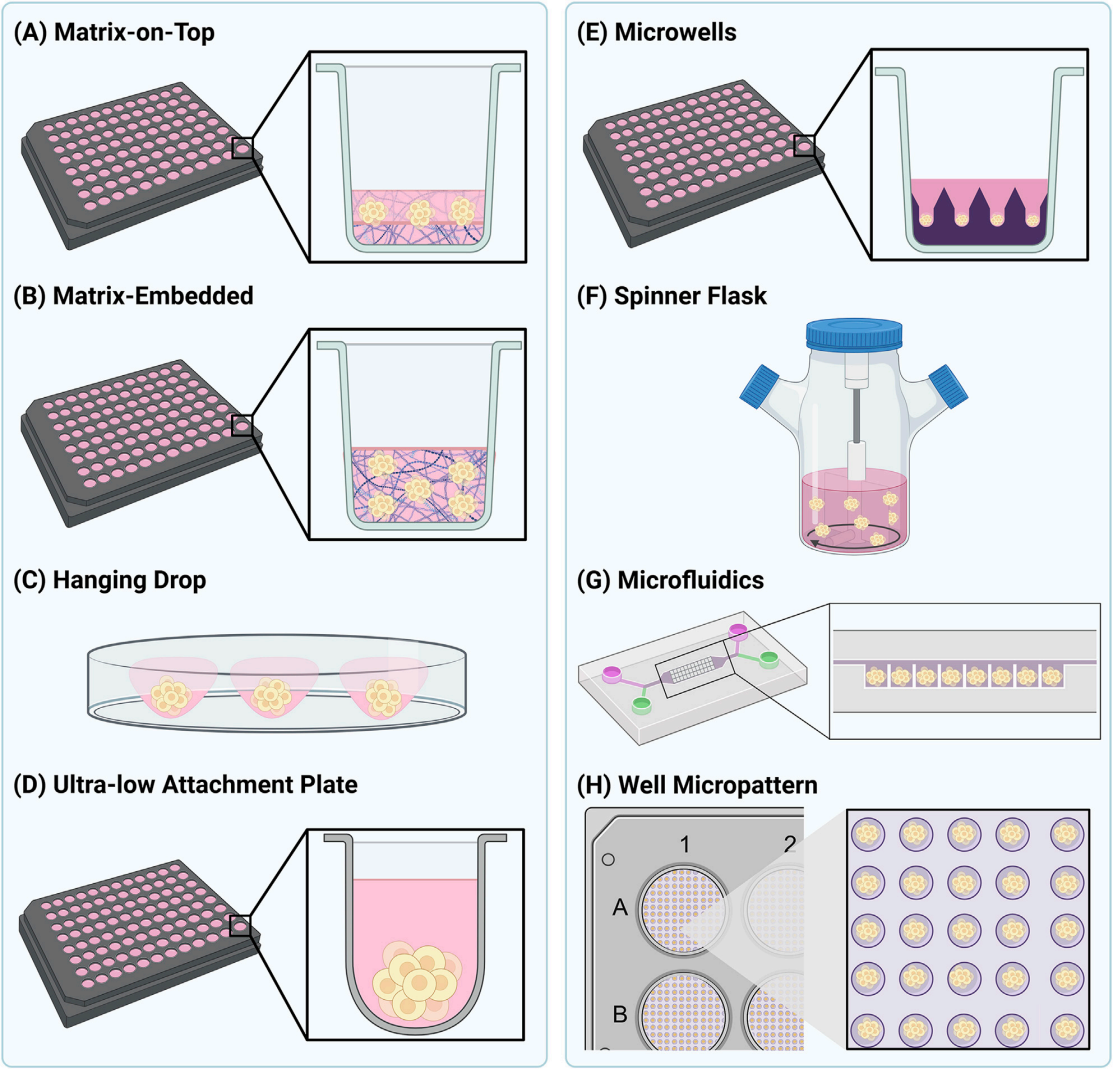
Schematic representation of several commonly used 3D cell culture methods. Methods to generate spheroids as shown in **(A)** and **(B)** are generally considered as scaffold-based methods, whereas the methods shown in **(C–G)** are considered scaffold-free. The method shown in **(H)** is a hybrid between scaffold-based and scaffold-free.

In addition to the varieties of 3D cell models available, a wide number of methodologies exist for their assembly. These methods are often divided into scaffold-based methods (Figures 1A,B) and scaffold-free methods (Figures 1C–G), with the method chosen being determined by the cell type, nature of 3D structure needed, and imaging approach being employed. Scaffold-based methods, used for both spheroid and organoid production, involve the growth of cells in a 3D environment to facilitate cell-cell and cell-ECM interactions (Abuwatfa et al., 2024). Biocompatible inserts, membranes, polymer scaffolds or hydrogels can all be used as structures to facilitate cell aggregation and assembly (Abuwatfa et al., 2024). Of these, polymers are probably the most used material, as they offer a high degree of control in terms of porosity and stiffness to suit the needs of different cell types. In most instances, the purpose of the scaffold is to at least partially replicate the extracellular environment that the cells would encounter *in vivo*. The scaffold should offer properties similar to the ECM that surrounds cells, and so many scaffolds are based on molecules such as collagen, laminin, fibronectin, and other sulphated and glycosylated macromolecules. In addition, growth factors can also be present. All these natural molecules can enhance cell survival and viability (Nunes et al., 2019). Matrigel, and similar products, is the most widely used commercially available natural ECM polymer, extracted from the Engelbreth-Holm-Swarm mouse tumour. Cells can be grown on top of (Figure 1A) or embedded (Figure 1B) in Matrigel (Abuwatfa et al., 2024; Caliari and Burdick, 2016). While this allows a degree of flexibility in its use, Matrigel has several disadvantages. One issue associated with this scaffold is the batch-to-batch variability, due to varying protein composition ratios (Van Zundert et al., 2020). Another challenge with Matrigel is that it is liquid at 4°C and gelates at 10°C, posing a challenge for its use in high-throughput and automated settings. High concentrations of Matrigel can also give rise to optical aberrations when imaging. However, these limitations are beginning to be tackled, and our own lab has previously described an optimised methodology for routine production of spheroids at a scale compatible with high-throughput automated fluorescence imaging. Such an approach would facilitate the systematic study of NP uptake into spheroids. Hydrogels are the most utilised artificial scaffold mimicking the ECM (Caliari and Burdick, 2016). Hydrogels are very flexible with regard to their assembly and adaptability for different biological questions (Ruedinger et al., 2015), and they can be used for production of spheroids through embedding methods (Figure 1B). In addition, a number of manufacturers have developed multi-well plates in which hydrogel materials have been used to create up to several hundred microwells per well (Figure 1E). In this context the hydrogel is rather acting as a tool to guide the position of a spheroid in a well, rather than provide an ECM scaffold that would fully encapsulate the growing spheroid. Such systems potentially provide exciting opportunities for screening of large populations of highly consistent spheroids and organoids (Decembrini et al., 2020; Lucendo-Villarin et al., 2020). Hybrid scaffolds for spheroid production have started to emerge, consisting of synthetic and natural polymers, thereby combining the advantages of both (Ferreira et al., 2018). While such scaffold-based technologies offer distinct advantages for NP uptake and distribution assays, it remains unclear as to whether the presence of such matrix material may influence NP behaviour. This should be systematically evaluated prior to any NP types moving on to the subsequent steps of clinical approval.

Scaffold-free culture techniques have also been employed to generate 3D cell structures (Foglietta et al., 2020). These methods rely on generating a low adhesion environment to encourage 3D cell growth, and are predominantly used to generate spheroids (Lv et al., 2017; Valdoz et al., 2021). In general, these methods rely on the intrinsic properties of cells to aggregate and self-assemble into higher-order 3D structures. As with scaffold-based methods, a variety of methodologies to achieve this are widely utilised (Foglietta et al., 2020). The hanging drop method (Figure 1C) relies on gravity to encourage the aggregation of cells into spheroids in a droplet of medium. Once the spheroid has reached the desired size, it can be transferred into a receiving plate for downstream experiments and analysis. An alternative approach is to grow the spheroids in a carrier that minimises cell interactions with the base, typically achieved using ultra-low attachment (ULA) plates (Figure 1D). Cells grown in ULA plates are unable to grow as traditional monolayers, and thus aggregate and assemble on the hydrophobic, or agarose-coated surfaces. This method is simple to use and produces uniform and reproducible spheroids in as little as 3 days, making it highly adaptable for high-throughput applications (Valdoz et al., 2021). Microwell arrays provide increased throughput compared to ULA plates and increased control over spheroid size (Lee et al., 2016). Microwells can be created using a concave poly (dimethylsiloxane) (PDMS) stamp, which is used to imprint microwells into a hydrogel such as poly (ethylene glycol) (PEG) (Figure 1E) (Lee et al., 2018). The shape and depth of the microwells can be altered to control spheroid size and the number of spheroids produced per well (Lee et al., 2023). Agitation of cells can also be used to produce 3D cellular structures by continuously stirring cells and promoting self-aggregation (Figure 1F). Many different devices are now available to facilitate this method; however, the agitation parameters need to be chosen carefully to avoid cell damage (Lv et al., 2017; Nunes et al., 2019). Customised microfluidic devices have also been adapted for producing spheroids, allowing them to grow in defined patterns and under continuous perfusion (Figure 1G) (Ko et al., 2024). These systems can be used to examine the influence of fluid dynamics on NP uptake and penetration in spheroids which are entrapped in the microfluidic chamber (Van Zundert et al., 2020). Both scaffold-based and scaffold-free spheroid culture systems can also be employed at the same time. This allows for the high numbers of spheroids produced in scaffold-based systems and the uniformity of shape and size afforded by scaffold-free systems to be combined. For example, this can be achieved in a high-throughput manner using micropatterned plates coated with fibronectin to produce hundreds of highly uniform spheroids per well (Figure 1H) (Mysior and Simpson, 2024).

The range of methods now available to culture spheroids and organoids has revolutionised our thinking around their use for studying NP-cell interactions. While each method has its own advantages and disadvantages, the key is that the one chosen can provide scale and consistency of spheroid or organoid production, and that it is compatible with the imaging approach planned. Indeed the issue of reproducibility is now beginning to be addressed, and efforts are underway for standardisation of organoid models including sample collection, culture conditions and the type of ECM material used (Zhou et al., 2023). Another example is the publication of guidelines for manufacture and application of organoids from stem cells, providing information on source cells, culture conditions, quality requirements and evaluation, and storage and preservation conditions (Ahn et al., 2024). There are also studies directly addressing the development of organoid-specific guidelines (Wang et al., 2025), spheroids (Lee et al., 2018) and standardisation of culture conditions (Zhu et al., 2025). Altogether, it is likely that standardisation will become increasingly important, particularly in the context of therapeutic testing.

## 3 Quantitative fluorescence microscopy

Mechanistic studies of NP-cell interactions necessitate the use of quantitative approaches. In this context, techniques, such as Fluorescence-Activated Cell Sorting and fluorescence microscopy have been widely adopted (Dzyhovskyi et al., 2024). Flow cytometry offers the distinct advantage of quantifying NP uptake and toxicity across large cell populations. While recent studies have demonstrated the visualisation of NPs in flowing cells using a technique called tomographic flow cytometry, this approach generally does not provide meaningful insights into NP intracellular distribution and is limited to examining cells in isolation (Pirone et al., 2021). In contrast, fluorescence microscopy is a highly versatile tool that enables the visualisation of dynamic processes across multiple scales, from whole organisms to individual cells and molecules, in both living and fixed specimens. The growing variety of fluorophores compatible with biological samples now allows for the simultaneous monitoring of multiple events, with numerous labelling techniques available to target structures of interest (Minoshima et al., 2024). In the case of NPs, a wide range of organic fluorescent dyes can be used, either encapsulated within the particle or, more commonly, covalently grafted to appropriate ligands on the particle surface. The addition of fluorophores to NPs enables the study of their spatial and temporal interactions with cells. Most excitingly, bio-responsive fluorophores in the near-infrared spectrum can be used in both *in vitro* and *in vivo* studies (Wang et al., 2020b; Zhang et al., 2012).

To visualise fluorescently labelled NPs and the cellular structure of interest, selecting the most appropriate microscopy technique is crucial (Gupta et al., 2024). This choice is largely determined by the type and size of the 3D cell assembly, as well as whether live imaging is required. Confocal microscopy has been a cornerstone of imaging for decades, offering optical sectioning of 3D samples. Though various methods exist to generate confocal images, the core principle involves using a pinhole to block out-of-focus light that originates above or below the focal plane from reaching the detector. In laser scanning confocal microscopy (LSCM), a laser point source excites fluorophores as it scans across the sample, with a single pinhole ensuring the exclusion of out-of-focus light. While this approach delivers high-quality images, its primary limitation is its relatively slow acquisition speed.

The spinning disk confocal microscope (SDCM) addresses this limitation by employing spinning disks with microlenses and pinholes arranged in an Archimedean spiral, enabling rapid illumination of the entire sample. Both LSCM and SDCM perform optical sectioning, a critical feature for imaging spheroids and organoids, but SDCM is generally favoured due to significantly faster imaging and reduced photobleaching. In both techniques, optical z-slices can be stacked to facilitate volumetric reconstruction of the spheroid or organoid. Conventional confocal microscopy is constrained by sample thickness, with high-resolution imaging typically limited to depths below 300 μm, depending on the optical system. This limitation can be partially addressed using two-photon or multi-photon microscopy (Luu et al., 2024; Palikaras and Tavernarakis, 2012; Xu et al., 2024). This technique uses powerful pulsed titanium sapphire or neodymium-doped yttrium lithium fluoride (Nd:YLF) lasers to excite fluorophores with two or more higher-wavelength photons simultaneously. Despite requiring specialised equipment, it enables deeper optical penetration (potentially greater than 1 mm), ideal for imaging large cellular assemblies (Palikaras and Tavernarakis, 2012). An alternative to LSCM and SDCM is light-sheet fluorescence microscopy (LSFM), which uses a defined light sheet to illuminate the sample. The separate illumination and detection paths allow for flexible shaping and positioning of the light sheet. While LSFM generally offers lower resolution than confocal microscopy, it shares key advantages, including optical sectioning of thick samples, reduced phototoxicity, and rapid image acquisition (Huisken and Stainier, 2009; Pesce et al., 2024).

Given the size of NPs, optical resolution is a key consideration when selecting an imaging modality. Super-resolution microscopy allows imaging beyond the diffraction limit of light, offering exciting possibilities for more precise NP localisation at subcellular resolution. Super-resolution images can be obtained through various methods, such as improved optics, structured illumination, or single-molecule localisation, each with its own advantages and limitations (Gupta et al., 2024; Salonen et al., 2008; Tantra and Knight, 2011). Nevertheless, all the above manual imaging methods are limited by low throughput. To address this, high-content screening (HCS) microscopy has been developed to enable large-scale acquisition of confocal imaging data. By automating the imaging process, HCS microscopy is particularly effective for applications such as cell-based drug screening, nanoscience, and drug delivery (Brayden et al., 2015).

Regardless of the imaging approach, it is widely recognised that image data must be quantified objectively and reproducibly. Typically, this begins with identifying objects of interest (e.g., nuclei, cellular structures, or NPs) in a process called segmentation. While segmentation algorithms are highly advanced for analysing cells in monolayers, volumetric segmentation and analysis of 3D cell assemblies remain significantly more challenging. Nonetheless, recent reports suggest that the gap between accurate 2D and 3D segmentation is narrowing (Hollandi et al., 2022; Piccinini et al., 2020; Salvi et al., 2019).

After object segmentation, the structures of interest must be quantified, with many possibilities and approaches to choose from. Absolute measurements include object count, volume, spatial distribution, and fluorescence intensity. Extracting such data at the subcellular level from 3D assemblies offers valuable insights into cellular organisation (Mysior and Simpson, 2024). When analysing NP distribution in spheroids, such values can be used to quantitatively assess the number of NPs in specific regions of the spheroid or cell, their co-localisation with cellular structures of interest, or their impact on a particular target. In addition to absolute measurements, descriptive metrics such as texture or morphology features are also valuable, especially when expected phenotypes or effects are heterogeneous. This form of analysis is increasingly utilised in drug development strategies (Esner et al., 2018), and as cell models become more complex, high-content analysis (HCA) approaches will undoubtedly be required to quantitatively characterise the observed effects.

Numerous open-source and commercial software tools are available to support quantitative image analysis. These tools are constantly evolving, and increasingly include artificial intelligence (AI) to extract data at the single-cell level (Carreras-Puigvert and Spjuth, 2024; Lacalle et al., 2021; Menduti and Boido, 2023; Novo et al., 2018; Shariff et al., 2010). Convolutional neural networks, including architectures such as U-Net, HRNet, and DeepLabV3+, have been shown to enable accurate, automated segmentation of complex structures such as spheroids and organoids (Lacalle et al., 2021; Oudouar et al., 2025). These tools support single-cell level feature extraction and phenotypic profiling, improving throughput and reproducibility. Both open-source and commercial platforms are increasingly integrating AI to streamline analysis workflows, reduce user bias, and handle large-scale datasets. Such AI-driven approaches are transforming how cell-cell interactions are analysed in increasingly physiologically-relevant systems (Dave et al., 2025; Lacalle et al., 2021; Piccinini et al., 2023; Oudouar et al., 2025).

Even so, as discussed in more detail below, relatively few imaging-based studies of NP uptake and penetration into cells have been quantitative. It is important to note that such studies are not without challenges. Fluorophores coupled to NPs can be sensitive to local environmental conditions and may dissociate in certain biological fluids, complicating their quantifications (Simonsen and Kromann, 2021).

## 4 Cellular uptake of NPs

**FIGURE 2:**
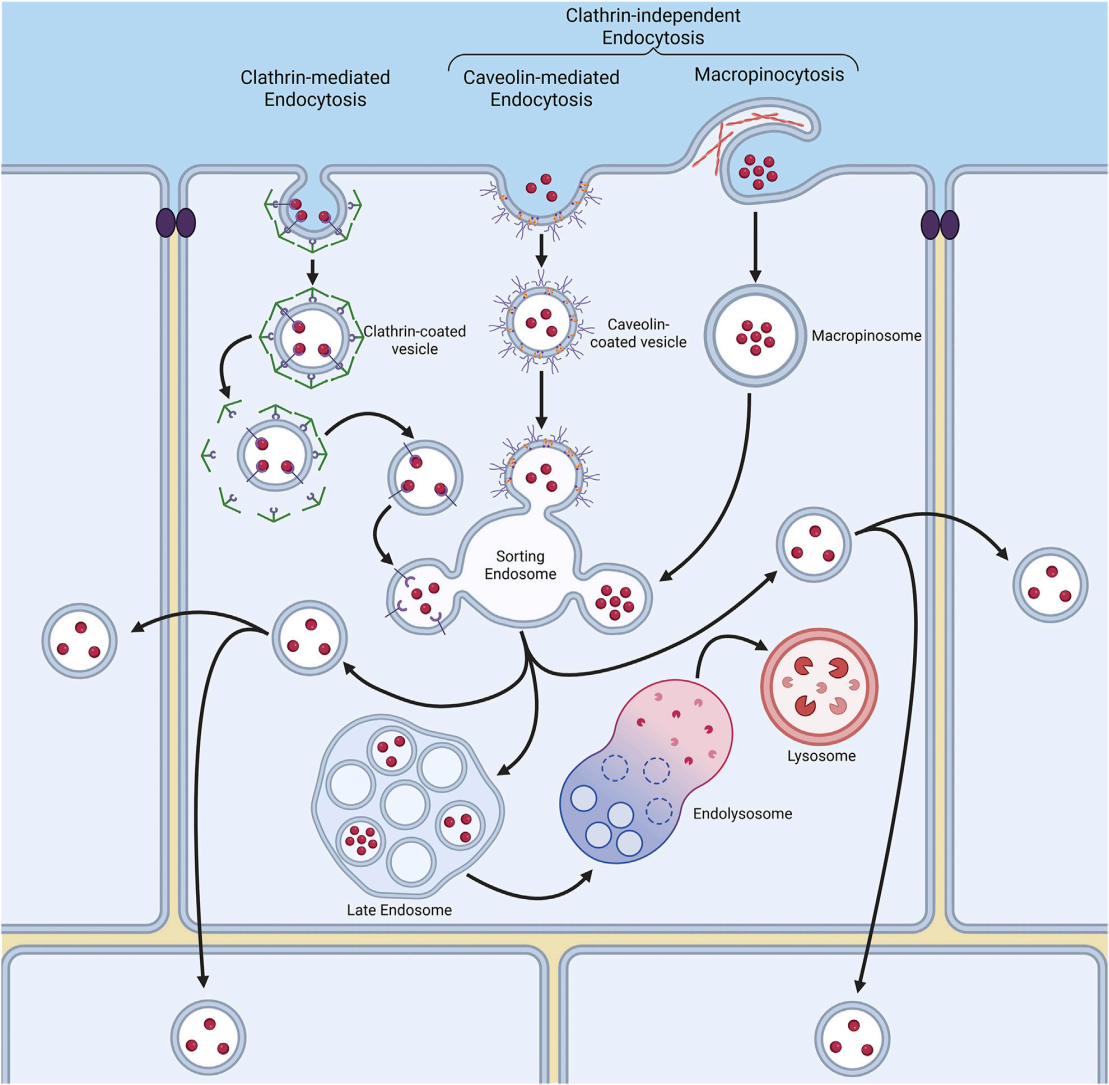
Endocytosis is a process by which cells internalise molecules through various membrane pathways. These pathways include clathrin-mediated endocytosis (CME), and clathrin-independent mechanisms such as caveolae-dependent endocytosis, macropinocytosis and phagocytosis. Each pathway involves distinct membrane remodelling events, such as the formation of clathrin-coated vesicles or caveolae, and dictates how nanoparticles (NPs) are internalised and trafficked within the cell to a variety of destinations, including transfer to other neighbouring cells.

Endocytosis is the process by which internal membranes are formed from the plasma membrane lipid bilayer, facilitating the internalisation of various molecules (Figure 2) (Kaksonen and Roux, 2018). The diversity of molecules internalised by a cell is achieved through distinct, highly regulated uptake pathways (Sigismund et al., 2021). The emerging picture is that NPs are endocytosed through various mechanisms (discussed in detail below), influenced by both NP properties and the cell model used (Garanina et al., 2024; Li et al., 2024; Zhao and Stenzel, 2018). Uptake mechanisms are broadly characterised into (1) clathrin-mediated endocytosis (CME) and (2) clathrin-independent endocytosis (CIE), which includes caveolae-dependent and -independent endocytosis, micropinocytosis, and phagocytosis (Figure 2). Clathrin-mediated endocytosis is driven by the assembly of a clathrin protein coat, which induces membrane curvature to form a clathrin-coated pit. A subsequent scission event at the plasma membrane results in the formation of clathrin-coated vesicles, which transport internalised cargo into the endomembrane system (Kaksonen and Roux, 2018). Caveolae-dependent endocytosis is mediated by the protein caveolin. Caveolae are flask-shaped, deeply invaginated structures that form from plasma membrane lipid rafts, primarily composed of glycosphingolipids, cholesterol, and GPI-anchored proteins (Sandvig et al., 2018). Upon scission, caveolin-containing vesicles are trafficked into the endo-lysosomal pathway, often delivering their cargo to an early endosome-like compartment known as the caveosome (Sandvig et al., 2018). Macropinocytosis and phagocytosis are specialised endocytic processes that internalise larger structures, involving major remodelling of the cell surface to create membrane outgrowths for cargo encapsulation (Sigismund et al., 2021; Uribe-Querol and Rosales, 2020; Wu et al., 2024). Understanding the mechanisms of NP endocytosis is crucial, as they determine the initial subcellular membranes which NPs encounter (Zhao and Stenzel, 2018). Furthermore, once internalised into cells, NPs have the potential to be channelled into a wide variety of subcellular pathways. Many of these pathways lead either to the acidic compartments of the endo-lysosomal system, or indeed back to the cell surface, which in both cases may result in poor therapeutic delivery outcomes. At the same time, pathways into neighbouring cells also exist, and NP entry into these routes offers exciting potential for the delivery of therapeutics deep inside a multi-cell layered tumour (Figure 2). With this in mind, and despite clear differences in NP-induced toxicity between 2D and 3D cell culture systems, surprisingly few studies have examined NP entry and transcytosis mechanisms in 3D models. Instead, research has largely focused on NP penetration into 3D cell assemblies, overlooking the specifics of cellular internalisation and subsequent endomembrane system traversal.

## 5 Imaging the cellular uptake of NPs

In the section above, we described how 3D cell assemblies are increasingly recognised as more suitable *in vitro* models for assessing drug delivery. However, before considering cellular effects following NP internalisation, these models may also play a crucial role in assessing the routes NPs take when first encountering cells. This issue is becoming increasingly relevant as nanomedicine research accelerates. As nanomaterials vary in material, shape, size, surface charge and functionalisation, it is now essential to determine the specific characteristics of each NP in relation to the nano-bio environment (Albanese et al., 2012; Habeeb et al., 2025; Lu and Stenzel, 2018). Currently, there are “sufficient deficiencies” of knowledge in this area (Gupta et al., 2024; Johnston, 2017), several reviews have underscored the importance of addressing these gaps emphasising the need for more comprehensive and detailed studies (FitzGerald and Johnston, 2021; Griffiths et al., 2022; Gupta et al., 2024; Johnston, 2017; Nelemans and Gurevich, 2020).

Traditionally, pharmacological inhibitors have been the most commonly used method to study NP internalisation pathways. In monolayer cell cultures, these studies can be quantified using fluorescence microscopy, such as co-localisation assays with organelle markers or through flow cytometry after detaching cells from the culture dish (Amar-Lewis et al., 2024; Sahay et al., 2013; Selby et al., 2018; Yap et al., 2024). Although widely used, chemical inhibitors of endocytic mechanisms often yield conflicting results due to their lack of specificity for a single pathway. An alternative is to employ RNA interference (RNAi) to selectively deplete proteins involved in endocytosis (Panarella et al., 2016; Pereira et al., 2015; Wang et al., 2017). In addition, genome-wide forward genetic screening approaches are also now being applied to more systematically identify the factors associated with NP uptake into monolayer cells (Montizaan et al., 2024). While RNAi tools provide high specificity, their potential remains underutilised in nanomedicine design. For absolute specificity, nanomedicines must target not only a specific cell type but also precise intracellular locations, making insights from RNAi screens highly valuable. However, there are a number of challenges associated with the use of RNAi in 3D spheroid models which should be considered. Ensuring uniform delivery of small interfering RNAs (siRNAs) to cells is particularly challenging in intact 3D models, as failure to do so can lead to variable knock-down of the target gene throughout the spheroid (Morgan et al., 2018; Pelofy et al., 2021; Riching et al., 2024). To achieve higher accuracy and complete depletion of gene activity, clustered regularly interspaced short palindromic repeats (CRISPR)/Cas9 can be used as an alternative to RNAi. This technique has seen limited use thus far in 3D systems, although there are a few examples of successful applications (Haase-Kohn et al., 2022; Matano et al., 2015; Schwank et al., 2013). CRISPR has some advantages over RNAi, including reduced off-target effects and more uniform silencing of gene activity across the spheroid (Gopal et al., 2020; Wade, 2015). However, CRISPR faces similar limitations to RNAi due to difficulties delivering charged, non-viral gene delivery vectors through a hydrogel matrix, and variable efficiency of sgRNAs and Cas9 activity (Gopal et al., 2020, Wade, 2015).

Another consideration is that nanomedicines intended to deliver payloads to the cytosol should be designed to evade the destructive low pH environment of the endo-lysosomal system (Rathore et al., 2019). Most quantitative uptake studies have focused on monolayer cell cultures. As an example, Daniele and colleagues studied the co-localisation of folate-targeted gold NPs with lysosomes in kB cells quantitatively with confocal live-cell imaging techniques. Their findings demonstrated that the surface density of targeting ligands on NPs influenced how efficiently they were taken up by cells and trafficked through the endo-lysosomal pathway - key steps that ultimately affect NP delivery and therapeutic payload release (Daniele et al., 2023).

As discussed above, monolayer models lack many key characteristics of human tissues, highlighting the need to shift more studies to 3D models for more accurate representation (Zhou et al., 2024). Below, we will outline a number of studies that have utilised fluorescence microscopy in 3D cell models, all aiming to deepen our understanding of NP-cell interactions and uptake mechanisms. Due to space constraints it is not possible to detail every piece of research reported, however additional studies are also summarised in Supplementary Table S1. The first studies addressing this paucity of information utilised chemical inhibitors of endocytosis. Using pancreatic multicellular tumour spheroids (MCTS), the uptake of NPs in the size range 30–100 nm was shown to be inhibited by filipin (Lu et al., 2015) and sodium azide (Durymanov et al., 2019), suggesting that uptake was an active, energy-dependent process involving caveolae. Both studies employed LSCM to visualise NP internalisation into the cells within the MCTS, although different quantification methods were used. Lu and colleagues measured the fluorescence intensity of the supernatant to determine whether water-soluble block copolymer micelles had been endocytosed (Lu et al., 2015). In contrast, a more recent study by Durymanov and colleagues utilised image analysis software to quantify the fluorescence intensity of polystyrene NPs within spheroids (Durymanov et al., 2019). However, neither study provided single-cell level analysis. Pancreatic spheroids were also employed to investigate the uptake of dendrimer-camptothecin NP conjugates labelled with Cy5 (Wang et al., 2020a). Their chemical perturbation of choice was the tyrosine kinase inhibitor, genistein, with LSCM employed to assess the distribution of NPs, smaller than 20 nm, throughout the MCTS. Qualitative analysis suggested that genistein significantly abrogated NP transfer to the inner cells of the spheroid, leading the authors to propose caveolae-mediated mechanisms were involved. However, contradictory findings have been reported using other pharmacological inhibitors of endocytosis. For instance, in malignant mesothelioma spheroids, uptake of paclitaxel-loaded expansile NPs was most strongly inhibited by the PI-3 kinase inhibitor wortmannin, whereas genistein had no effect (Lei et al., 2015). This study nevertheless concluded that NP endocytosis in MCTS is energy-dependent, suggesting macropinocytosis as the primary mechanism for NPs larger than 100 nm. Such particle sizes exceed the upper limit of interaction with caveolae endocytic machinery.

NP size undoubtedly has a major influence on uptake efficiency and distribution in spheroids (Durymanov et al., 2019). Collectively, these studies do not yet provide a clear understanding of the endocytosis mechanisms utilised by NPs to enter cells in 3D assemblies (Nelemans and Gurevich, 2020). While many studies suggest caveolae-mediated pathways predominate, these conclusions often rely on genistein as the inhibitor of choice. Genistein, a naturally occurring isoflavone, is thought to have multiple molecular targets inside cells, and its activity is not specific to inhibition of caveolae-mediated endocytosis. Indeed its wide range of targets has seen the molecule being increasingly used as a therapeutic in its own right (Shete et al., 2024). Given the growing recognition of the lack of specificity in such chemical inhibitors (Griffiths et al., 2022), conclusions drawn from these studies should be interpreted with caution. We propose that a deeper understanding of the endocytosis mechanisms used by NPs to enter spheroids requires the application of precise molecular tools such as RNAi. Several studies in monolayer cells have utilised RNAi to deplete key endocytic proteins, including clathrin, caveolin-1, and flotillin-1, representing distinct endocytic pathways. While these studies in 2D cultures provide some insight into mechanisms that might operate in 3D assemblies, the picture even in 2D systems remains unclear. For instance, RNAi-mediated depletion of clathrin heavy chain or caveolin-1 in HeLa cells showed that 40 nm polystyrene NPs are preferentially endocytosed via CME, whereas larger 150 nm NPs are taken up via caveolae (Wang et al., 2017). These findings conflict with spheroid studies using chemical inhibitors. Again in HeLa cells, larger (>200 nm) chitosan nanogels were found to be internalised via flotillin-1- and Cdc42-dependent pathways, mechanisms better suited for larger particles (Pereira et al., 2015). In another RNAi study, small lipid NPs were primarily endocytosed via CME and macropinocytosis in HeLa cells (Gilleron et al., 2013), yet Sahay et al. (2013) observed no impact of clathrin heavy chain depletion on lipid NP uptake. Thus, the mechanisms of NP internalisation remain unclear in both monolayer cells and spheroids. Similar to NP-induced toxicity responses, it is reasonable to assume that different endocytosis mechanisms may operate in 2D versus 3D cell growth environments. Identifying and controlling these mechanisms is critical for nanomedicine design, as *in vivo* NP targeting specificity is determined by the ligand-receptor interactions between NPs and cells (Basinska et al., 2021; Colaço et al., 2020). We advocate for a large-scale, systematic analysis of NP endocytosis across a range of NP types and sizes. Such studies should incorporate single-cell-level data from within spheroids to comprehensively elucidate the mechanisms of NP uptake in 3D environments.

## 6 NP trafficking in spheroids

While the uptake mechanisms of NPs in 3D cell cultures are poorly understood, even less is known about the intracellular trafficking pathways they follow once internalised. Understanding this aspect of NP behaviour is critical for drug delivery, as therapeutics often need to reach specific subcellular locations to achieve their desired effect (Yameen et al., 2014). In a 3D context, of critical importance is that NPs must also access transcytosis pathways to penetrate cells located deep within tightly packed structures. Transcytosis is an active, energy-dependent process in which endothelial cells reorganise their cytoskeleton and plasma membrane to facilitate the movement of macromolecules from one face of a cell to another. This process involves the formation of endocytic vesicles that can internalise NPs, and then deliver them to the opposite side of the cell, where exocytosis will then release the NP making it available to be endocytosed into the next cell, deeper in the tumour (Sindhwani et al., 2020). This is essential, as studies have shown that NPs cannot generally pass between cells in dense 3D assemblies (Margalef-Català et al., 2019). Instead, they must utilise intracellular and transcytosis pathways to reach central regions. For NPs lacking specific subcellular targeting signals, endocytosis typically directs them to the endosomal-lysosomal pathway (Behzadi et al., 2017). A recently reported forward genetic screen, employing a retroviral gene trap in monolayer-grown HAP1 cells (Llargués-Sistac et al., 2023), identified 80 genes enriched in terms of playing a role in intracellular NP accumulation (Montizaan et al., 2024). Information from such studies will be important in defining the critical machinery that determines NP fate in cells, and whether their entry into the terminal lysosomal pathway can be avoided, a key factor in the design of nanomedicines for use in solid tumours where they need to be able to cross through multiple cell layers.

Fluorescence microscopy has been widely used to study NP trafficking, with co-localisation techniques providing insights into their interaction with membrane compartments. Studies in monolayer cells have employed various approaches, including fluorescent sensors (FitzGerald et al., 2020; Selby et al., 2018) and fluorescently tagged proteins marking endomembrane system compartments (Aoyama et al., 2017; Sahay et al., 2013; Sandin et al., 2012; Vtyurina et al., 2021; Zhang et al., 2016). While these studies offer quantitative data on the subcellular distribution of NPs, they do not directly elucidate the mechanisms underlying NP transport.

Functional and quantitative analyses of NP transport are more effectively achieved using HCS microscopy. To date, only two large-scale microscopy screens have systematically identified cellular machinery involved in NP trafficking through the endosomal-lysosomal system, both performed in monolayer cultures. Our lab previously conducted one such study using RNAi to downregulate 408 genes associated with cytoskeletal and membrane functions, employing small interfering RNAs. Automated imaging combined with HCA was employed to quantify the delivery of 40 nm carboxylate-modified polystyrene NPs to lysosomes. This study highlighted the involvement of various endocytic regulators, including CME machinery, and notably identified roles for membrane remodelling proteins such as Rab33b and OATL1, as well as the molecular motor myosin VI (Panarella et al., 2016). More recently, Ross-Thriepland and colleagues conducted an arrayed CRISPR screen to investigate the uptake and trafficking of lipid NPs encapsulating an mRNA encoding the fluorescent protein mCherry. This approach used mCherry as a reporter for the successful delivery of lipid NPs into cells. Screening over 7,000 genes from the druggable genome, the study identified and validated 44 candidates that either enhanced or inhibited lipid NP uptake and trafficking. Among these, two genes emerged as particularly noteworthy. Deletion of UDP-glucose ceramide glucosyltransferase, a key enzyme in glycosphingolipid biosynthesis, was found to enhance lipid NP delivery. Conversely, deletion of the V-type proton ATPase (ATP6V) abolished LNP-mRNA uptake (Ross-Thriepland et al., 2020). Although this study provided limited details about the lipid NPs, such as their size, it underscored the potential of NP-based systems for targeted delivery. These findings highlight the power of systematic approaches to uncover the mechanisms governing NP trafficking through the endomembrane system. However, applying these techniques at scale in 3D cell models remains an undeniable challenge.

Efforts to investigate the subcellular pathways taken by NPs in 3D cell models are however beginning to emerge. These studies hold significant promise, as they could not only elucidate how NPs traverse intracellular compartments within a single cell but also, unlike equivalent experiments in monolayer cells, reveal how NPs transfer between adjacent cells. A molecular understanding of the transcytosis pathways that enable NPs to move from one cell to another is essential to advance therapeutic strategies (Jang and Sengupta, 2019; Zhou et al., 2022). Transcytosis has been suggested as the mechanism for polystyrene NP transfer in pancreatic spheroids, although this was not explicitly demonstrated (Durymanov et al., 2019). Further evidence supporting transcytosis as a means of NP transit between cells comes from two spheroid-based studies that utilised LSCM to examine cells treated with the small molecule Exo1 (Lu et al., 2015; Wang et al., 2020a). Exo1 induces the collapse of the Golgi apparatus into the endoplasmic reticulum, thereby rapidly inhibiting the secretory activity of cells (Feng et al., 2003). Notably, both studies observed similar reductions in NP penetration into central cells within the spheroids, despite using different NP types (polymeric micelles and a dendrimer-camptothecin conjugate) and different pancreatic cell lines in their models - AsPC-1 and BxPC-3 cells, respectively (Lu et al., 2015; Wang et al., 2020a). These findings highlight a conserved role for transcytosis in facilitating NP transport in 3D cell models. These two studies establish an excellent foundation, utilising LSCM, for further exploration of NP intracellular transport. However, a critical next step will be to make these analyses quantitative and at a higher optical resolution to obtain detailed subcellular information. The regulation of transcytosis is inherently complex, requiring precise sorting of cargo at multiple junctures along the endocytic and exocytic pathways. We propose that RNAi is a powerful tool for dissecting these processes at the molecular level, particularly by targeting key molecules such as the Rab family of small GTPases, which act as master regulators of membrane trafficking pathways. Preliminary experiments in HT-29 colon cell spheroids have demonstrated the feasibility of this approach. By targeting a subset of Rab proteins and quantifying co-localisation of polystyrene NPs with subcellular markers using HCS and HCA, these studies highlight the potential of RNAi to unravel the molecular basis of NP transcytosis (Cutrona and Simpson, 2019). Although such studies are in their infancy, they underline the exciting possibility of performing a detailed molecular dissection of NP transcytosis in 3D cell models using advanced quantitative imaging techniques.

## 7 NP penetration into 3D cellular assemblies

A critical parameter for quantification in 3D models is the efficiency of NP penetration into the central regions of the structure. Understanding NP distribution is vital with respect to the ultimate goal of optimising the therapeutic efficacy of any nano-based drug delivery system *in vivo* (Jain and Stylianopoulos, 2010). There are a number of barriers which prevent the delivery of NPs to the centre of solid tumours, including mucosal barriers, endothelial barriers, and the local arrangement of the extracellular matrix (Shen et al., 2024). Incorporating these features into a 3D model is challenging, although one recent study has employed use of a U87-MG glioblastoma xenograft model, combined with both electron microscopy and CLSM, to study gold NP penetrative ability (Sindhwani et al., 2020). Although this work did not assess the molecular mechanisms governing NP transport through a solid tumour, it made the important conclusion that this process was active and required cell-to-cell transfer of the NPs, rather than them passing through gaps between cells. Methodologies to understand the basis of this penetration into 3D cell assemblies are now gaining in popularity, and clearly the direct visualisation of fluorescently-labelled NPs by CLSM, SDCM or other imaging techniques offers huge potential to further our understanding of NP dynamics in a relevant spatial context. In this regard, a growing number of studies have employed 3D models of different cell types, representing various tissues associated with tumours, including colon, breast, pancreas, lung, and brain. Examples of each of these are discussed briefly below. It should be noted that while the use of various imaging modalities is the common link between each of the studies mentioned in these next sections, individual cellular or subcellular information was not assessed in any of them, highlighting our lack of knowledge in this area.

### 7.1 Colon

Cells derived from colon tissue were among the first cell types utilised for 3D studies, with the choice being influenced by both the relevance of this tissue from a therapeutic delivery perspective and also the levels of occurrence of colon tumours. Colon spheroids assembled from HCT116 cells have been used in several studies to investigate the ability of NPs with different surface properties and different sizes to penetrate through a 3D environment (Rane and Armani, 2016; Tchoryk et al., 2019). Work with polystyrene NPs of 30 nm and 50 nm in diameter displayed enhanced penetration into spheroids in comparison to that of 100 nm NPs (Tchoryk et al., 2019). A similar phenotype was also seen for spherical gold NPs of 30 nm, in comparison to 50 nm and 70 nm NPs (Rane and Armani, 2016). Additionally, the shape of NPs plays a role in their ability to penetrate into spheroids. In comparison to spherical gold NPs, rod-shaped gold NPs were unable to penetrate as deep into HCT116 spheroids (Rane and Armani, 2016). Common to both studies was the use of microscopy to calculate the penetration of the different NPs, thereby providing the ability to visualise the location of NPs within a spheroid. In this regard, the type of microscopy used can be critical, as different modalities offer different light penetration properties. For example, Tchoryk and colleagues quantified the distribution of the different size NPs within the different areas of the HCT116 spheroid (periphery, middle and core) using LSCM (Tchoryk et al., 2019), whereas Rane and Armani (2016) used two-photon photoluminescence microscopy (Rane and Armani, 2016). The latter technique is potentially more favourable for analysing 3D objects such as spheroids due to increased depth of light penetration into the sample (Costa et al., 2016).

### 7.2 Breast

A number of breast cancer cell lines have been used to study the penetration of several NP formulations into spheroids such as, gold NPs (Chen et al., 2024), quantum dots (Jarockyte et al., 2018), mesoporous silica NPs (MSNs) (Wang et al., 2024; Fang et al., 2024) and block copolymer micelles (Yu et al., 2021). A common theme emerging from these studies is the effect of NP size and shape on penetration. Fang and colleagues prepared spherical, rod-shaped, and hexagonal-plate-shaped MSNs to compare their ability to penetrate to the centre of tumour spheroids composed of 4T1 breast cancer cells. They observed that the hexagonal-plate shaped MSNs showed the highest penetration to the centre of the spheroids after 24 h, followed by the spherical MSNs, with the rod-shaped MSNs showing the lowest penetration levels, requiring 48 h to reach the centre (Fang et al., 2024). Another study utilised LSCM to examine the penetration of rod-shaped block copolymer micelles in both MDA-MB-231 and MDA-MB-436 breast cancer spheroids. They quantified penetration of the micelles by dividing the spheroids into equally spaced centric regions, namely, the periphery, intermediate and central areas, which allowed them to look at NP penetration across different parts of the spheroid. Using quantitative image analysis, they found that the micelles were more readily taken up by MDA-MB-231 spheroids than MDA-MB-436 spheroids, regardless of their length (Yu et al., 2021). MCF-7 spheroids have also been utilised to study NP penetration using more advanced microscopy techniques such as light-sheet microscopy (Chen et al., 2018) and super-resolution microscopy (Liu et al., 2020). Light-sheet microscopy enables deeper light penetration into the sample and is therefore favourable for imaging 3D spheroids (Costa et al., 2016). The use of super-resolution to study the penetration of upconversion NPs is particularly interesting. Liu et al. (2020) demonstrated that using near-infrared Bessel-beam emission saturation nanoscopy, single NP tracking within a 3D environment can be achieved. Although super-resolution approaches are not currently suited to high-throughput experiments and are thus time-consuming and expensive, this method provides an exciting opportunity to more precisely visualise NP uptake and trafficking events in 3D cell culture systems (Liu et al., 2020). Priwitaningrum and co-workers treated 4T1 mono-spheroids and 4T1:3T3 hetero-spheroids with polymeric micelles (PMCs) and polymeric NPs (PNPs) and imaged them using two-photon microscopy. To assess their penetrative ability, intensity profiles of the PMCs and PNPs were quantified across the spheroids. This revealed that both nanosystems penetrated deeper into the mono-spheroids than that of the hetero-spheroids, implicating the tumour stroma in the inhibition of NPs into the spheroid (Priwitaningrum et al., 2023). This is a striking example as to how quantitative fluorescence microscopy can provide in-depth insight into the ability of NPs to penetrate 3D spheroids.

### 7.3 Pancreas

The influence of size on NP penetration into spheroids composed of pancreatic cells has also been investigated (Durymanov et al., 2019; Priwitaningrum et al., 2023). Polystyrene NPs of 20 nm were found to penetrate deeper into both BxPC3 and PANC-1 spheroids, in comparison to 100 nm and 500 nm polystyrene NPs (Durymanov et al., 2019). Similarly, PANC-1 spheroids internalised 30 nm silica NPs (SiNPs) more readily than that of 100 nm SiNPs (Priwitaningrum et al., 2023). Both studies also used LSCM to visualise and subsequently quantify NP penetration into pancreatic spheroids. Another pancreatic cancer cell line, AsPC-1, has been used to study crosslinked and uncrosslinked polymeric micelles (Lu et al., 2015). LSCM revealed that penetration of the different types of micelles was limited to the periphery of the spheroid (Lu et al., 2015). PANC-1 and pancreatic stellate cells have been co-cultured to form hetero-spheroids (Darrigues et al., 2020). These hetero-spheroids were then employed to look at the penetration of functionalised gold nanorods (AuNRs), and the role of the stromal cells in limiting AuNR penetration using a combination of fluorescence, photoacoustic and photothermal microscopy. In comparison to PANC-1 homo-spheroids, which enabled penetration of AuNRs more readily, hetero-spheroids displayed only peripheral penetration of AuNRs (Darrigues et al., 2020). Lazzari and colleagues utilised a triple co-culture of PANC-1, MRC-5 and HUVEC cells to investigate the penetration of doxorubicin-loaded NPs throughout the solid hetero-spheroid. Both LSCM and LSFM were compared for their ability to visualise NP penetration and they found that LSFM provided superior imaging of structures with a depth greater than 100 µm (Lazzari et al., 2019). Additionally they observed that only free doxorubicin was able to penetrate to the centre of the hetero-spheroid, with doxorubicin-loaded NPs being unable to penetrate (Lazzari et al., 2019). The NPs used in this study were ca. 100 nm in size with a hydrophobic surface, which may have reduced their ability to diffuse through the ECM-rich pancreatic hetero-spheroids. This highlights the value of 3D cellular models for testing such therapeutics and the importance of size in dictating their ability to penetrate across layers of cells.

### 7.4 Lung

Lung cancer spheroids are particularly important models for studying NP penetration and transport, due to the ability of nanomedicines to be taken via inhalation (Bölükbas and Meiners, 2015). Lu and colleagues studied the role of surface properties in AuNP penetration using A549 spheroids. By measuring the fluorescence intensity of optical sections of the spheroid, it was found that modification of AuNPs with poly (2-hydroxylethyl acrylate) (pHEA) of longer length improved penetration (Lu et al., 2020). Interestingly, these results varied from those in monolayers, with medium length pHEA-AuNPs showing increased penetration. The penetration of iron oxide NPs (IONPs) in MCF-7 breast and H1229 lung spheroids has also been studied. It was found that IONPs loaded with doxorubicin penetrated uniformly throughout the spheroid in comparison to native doxorubicin (Basuki et al., 2013); a finding that differed from that described above in pancreatic cell models. A549 mono- and hetero-spheroids grown in co-culture with 3T3 fibroblasts have also been used to compare the penetration of free doxorubicin with doxorubicin that is conjugated to dendrimers (GS4A-GFLG-DOX), thus forming a nanocarrier system (Almuqbil et al., 2020). Using LSCM, the penetration of both doxorubicin and GS4A-GFLG-DOX was quantified based on the mean fluorescence intensity at the core of the spheroids. For both mono- and hetero-spheroids, the nanocarrier system showed significantly greater penetration than that of free doxorubicin (Almuqbil et al., 2020).

### 7.5 Brain

The use of NPs to carry therapeutics across the blood-brain barrier *in vivo* offers tremendous potential, particularly in the context of treating neurodegenerative diseases (Jiménez et al., 2025; Romero-Ben et al., 2025). As such, developing spheroid models from brain cells represents one important step towards realising this goal. The penetration of different types of lipid NPs has been studied in U87-MG MCTS (Cabral et al., 2011; Niora et al., 2020). PEGylated liposomes, liposomes, lipoplexes and reconstituted high-density lipoproteins (rHDL) were labelled with the fluorescent tag DiI and added to brain spheroids. Imaging was carried out using LSFM and penetration was measured by looking at the fluorescence intensity of NPs within the MCTS. This revealed that rHDL lipoproteins outperformed the other lipid NPs in terms of ability to penetrate to the centre of the spheroid (Niora et al., 2020). Furthermore, the rHDL lipoproteins were smaller than the other NPs assessed, and therefore the improved penetration of smaller NPs seen in this example is consistent with that described for other spheroid type penetration studies. Sokolova and colleagues also examined brain hetero-spheroids using human primary cells, specifically brain microvascular endothelial cells, astrocytes and pericytes to analyse uptake and penetration of various ultra small (ca. 2 nm) fluorescent AuNPs (dye-clicked (Au-Click-FAM and Au-Click-Cy3) and peptide-functionalised (Au-CGGpTPAAK-FAM)). It was found that all three types of AuNPs were internalised into the spheroids, whereas the dissolved dyes alone did not enter the spheroids. This is an interesting contrast to the findings of Lazzari and colleagues (2019), highlighting the variability in response between tissues and the importance of NP size for delivery. However, it is important to note that this was simply observational data with no quantitative analysis of NP penetration performed (Sokolova et al., 2020). Regardless of the missing quantification of the penetration of NPs into the spheroid core, this study is another example showcasing that NP size and spheroid model used are critical considerations when designing experimental setup.

## 8 Conclusion and future perspectives

The use of microscopy techniques to unravel nano-bio interactions in mammalian 3D cell models, such as spheroids and organoids, provides an unprecedented opportunity to advance our understanding of NP behaviour. These models undoubtedly present a more physiologically relevant context for studying NP uptake, trafficking and uniquely, penetration across cell layers, compared to traditional monolayer cultures. However, the full potential of microscopy, particularly in studying NP toxicity and efficacy in 3D models, remains underutilised. Furthermore, such studies have the potential to provide unparalleled insights into the subcellular effects of NPs, particularly their impact on organelles, which could inform the development of safer and more effective nanomedicines.

Despite progress in NP uptake and trafficking research, significant knowledge gaps persist - particularly in understanding the molecular mechanisms that govern NP endocytosis and trafficking within complex 3D environments. While classic pharmacological inhibitors have been widely used to probe these processes, their non-specificity often limits the precision of mechanistic insights. Nevertheless, some trends are beginning to emerge. For instance, smaller NPs, typically less than 50 nm in diameter, consistently demonstrate superior penetration into multicellular spheroids and tumour-like structures compared to their larger counterparts. The likelihood for this observation is that many intracellular vesicles, which are the carriers responsible for the movement of cargo between subcellular compartments, are also in the nominal size range of 60–80 nm. NPs of sizes greater than this may be able to be internalised into cells via mechanisms such as micropinocytosis, however, they may struggle to access the vesicles facilitating transcytosis pathways. The surface properties of NPs, such as charge and functionalisation, also play critical roles in modulating their uptake and intracellular routing. Advanced microscopy techniques, including LSFM, super-resolution microscopy, and multiphoton microscopy, are proving instrumental in revealing these dynamics. LSFM and multiphoton microscopy enable deep imaging of large 3D structures, whereas super-resolution microscopy provides nanoscale resolution crucial for visualising NP behaviour within subcellular compartments. However, the relatively high cost of these techniques and their limited compatibility with high-throughput workflows remain significant barriers to widespread adoption. Also of consideration, particularly as we try to develop more standardised studies to compare NP penetration ability, is the size of the spheroid model used. Very few of the studies presented in the literature to date use consistently-sized spheroids, making the kinetics of NP penetration very difficult to compare between NP types. Some of the emerging methods to produce highly consistent spheroids, described earlier in this review, should address this. Furthermore, more complex spheroids, such as multi-cell type hetero-spheroids, also move us closer towards using models that are of clinical relevance.

Future research should prioritise quantitative analyses of NP uptake, intracellular trafficking and toxicity using these advanced imaging modalities. Coupling such technologies with RNAi, CRISPR and other molecular tools will provide a deeper understanding of NP dynamics and the regulatory mechanisms governing transcytosis and intercellular transport. Additionally, expanding studies to include patient-derived tumour explants represents a potential step forward for the use of NPs in more personalised medical regimes. These models provide a more clinically relevant framework to study NP behaviour, offering insights into patient-specific responses and enabling the development of precision nanomedicines (Mui et al., 2023; Tieu et al., 2021). Combining patient-derived models with high-resolution microscopy and advanced imaging analytics could bridge the gap between preclinical findings and therapeutic application, dramatically improving the translational potential of nanomedicine research. However, this is not without its challenges. Simpler models, including spheroids and organoids, offer higher reproducibility for pre-clinical NP uptake studies, and are amenable to laboratory manipulation approaches. Explants, on the other hand, while providing exquisite insight into effective drug regimes, are notoriously heterogeneous, even at the level of those derived from an individual patient. This is perhaps one excellent example of where AI and machine learning are expected to play an increasingly prominent role in image analysis. These tools offer scalable solutions for segmentation and phenotypic profiling, enhance analytical depth and reproducibility, can be applied to highly diverse sample types, and potentially can save significant amounts of research time.

In conclusion, while significant progress has been made, fully realising the potential of NPs in medicine will require a concerted effort to leverage emerging imaging technologies, integrate patient-relevant models, and refine our understanding of the molecular mechanisms underpinning NP behaviour. Key bottlenecks include the challenges associated with producing 3D cell assemblies of high consistency at scale, overcoming optical limitations during imaging, and more efficiently and reproducibly extracting meaningful quantitative information at multiple scales from the assemblies. Such efforts will be worthwhile; not only advancing fundamental science but also accelerating the development of safe and effective nanomedicines tailored to the complexities of human biology.
