# Supplementary material for: Harnessing 3D cell models and high-resolution imaging to unveil the mechanisms of nanoparticle-mediated drug delivery
Source: Front Bioeng Biotechnol. 2025 Jul 7;13:1606573. doi: 10.3389/fbioe.2025.1606573 (PMC12277333; doi:10.3389/fbioe.2025.1606573)
Supplement: Supplementary file 1 [file Table1.docx]

***Supplementary Material***

# Supplementary Data

## Supplementary Table 1. Summary of various studies that used different imaging modalities to quantify nanoparticle trafficking within 3D cellular models.

| **Imaging Modality Used** | **Nanomaterial Size and Type** | **Cell Model** | **Observations / Comments** | **Reference** |
| --- | --- | --- | --- | --- |
| Brightfield Microscopy | ~ 370 nm polyethylene glycol-encapsulated Fe_2_O_3_​ NPs | HeLa and FaDu spheroids | Imaging revealed perinuclear NP localisation in 2D models, while in 3D spheroids NPs penetrated into hypoxic regions. NP-radiation interactions were model-dependent, with radio sensitisation in HeLa spheroids and radioprotection in FaDu spheroids. | Popescu et al., 2023 |
| Confocal Microscopy | ~ 50 nm copolymer micelles and ~ 260 nm DTX-loaded copolymer micelles | HeLa and HT-29 MCTS | Imaging of spheroids highlighted greater treatment resistance than in 2D models, linked to microenvironmental barriers. DTX-loaded micelles outperformed Taxotere®, showing improved efficacy in both models. | Mikhail et al., 2013 |
| Confocal Microscopy | ~ 30 nm crosslinked and un-crosslinked block copolymer micelles | AsPC-1 MCTS | Crosslinked micelles showed more efficient NP delivery than non-crosslinked ones, relying on transcellular transport rather than passive diffusion. Imaging in collagen-based spheroids showed that micelles accumulated mostly in the outer cell layers, suggesting limited penetration through dense extracellular matrix. | Lu et al., 2015 |
| Confocal Microscopy | 200 nm PLGA NPs,  30 nm and 70 nm Silica NPs | 3T3 and 4T1 homo- and hetero-spheroids, MDA-MB-231 and Panc-1 homo-spheroids, MDA-MB-231/BhJ-hTert and Panc-1/pancreatic stellate cells hetero-spheroids | Imaging showed that 30 nm NPs penetrated deeply (~75–80%) into cancer-only spheroids, while co-culture with fibroblasts significantly reduced penetration. Lower zeta potential also decreased uptake. Homo-spheroids allowed greater NP access than hetero-spheroids in both mouse and human models. | Priwitaningrum et al., 2016 |
| Confocal Microscopy | 20 nm, 50 nm and 100 nm polystyrene NPs | BxPC3 and Panc-1 homo-spheroids and Panc-1/3T3 hetero-spheroids | Imaging showed that 20 nm NPs penetrated BxPC3 spheroids via endocytosis and transcytosis, while larger particles were excluded. In PANC-1 spheroids, NP penetration also declined with size but was less dependent on active transport. | Durymanov et al., 2019 |
| Confocal Microscopy | ~ 5 nm TiO_2_  ~ 20nm Ag  ~ 150 nm ZnO | HepG2 spheroids | In HepG2 spheroids, Ag-NPs were less toxic than in 2D cultures, ZnO-NPs showed similar toxicity in both, and TiO₂-NPs were non-toxic. The 3D model better reflected NP behaviour, supporting its use for nanotoxicity screening. | Elje et al., 2020 |
| Confocal Microscopy | ~ 150 nm SiO_2_ | HepG2 spheroids (referred to here as microtissues) | SiO₂ NPs penetrated ~20 µm into pre-formed spheroids but only reached the core when added during formation. Penetration was limited by tissue structure and cell density, with no effect on spheroid size. | Fleddermann et al., 2019 |
| Confocal Microscopy | 30 nm, 50 nm, 100 nm unmodified polystyrene NPs  50 nm carboxylated, aminated variants  100 nm PEGylated NPs | HCT116 spheroids | Smaller poly(styrene) NPs (30 - 50 nm) penetrated HCT116 spheroid cores more efficiently than 100 nm NPs, with performance comparable to doxorubicin. Higher surface charge reduced penetration, while PEGylation enhanced core access. The spheroid flow cytometry model enabled precise comparison of NP design features. | Tchoryk et al., 2019 |
| Confocal Microscopy | ~ 90 nm and ~ 300 nm mesoporous silica NPs | MCF-7 spheroids | Continuous dosing enhanced mesoporous silica NP penetration in 3D spheroids. NP size had limited impact on uptake, but HAase pretreatment improved penetration of larger particles. | Zhuang et al., 2019 |
| Confocal Microscopy | ~ 3-4 nm AuNPs | hpBECs, hpPs and hpAs primary brain spheroids | Ultrasmall gold NPs (~3 - 4 nm) crossed the blood-brain barrier in 3D spheroids, with permeability increasing under hypoxic conditions. Imaging showed greater NP and dye uptake post-hypoxia, supporting their ability to access the brain microenvironment. | Sokolova et al., 2020 |
| Confocal Microscopy | ~ 20 nm AuNPs | HeLa and LNCaP spheroids | Combined treatment with docetaxel, AuNPs, and radiation significantly reduced cell survival and altered spheroid growth, indicating synergistic radio sensitisation and treatment-induced disruption of 3D tumour structure. | Bromma et al., 2023 |
| Confocal Microscopy | ~ 240 nm MSNs | 4T1 tumour spheroids | NP shape influenced penetration into 3D tumour spheroids and downstream therapeutic performance. Hexagonal-plate MSNs showed faster and deeper spheroid penetration than spherical or rod-shaped NPs, along with prolonged circulation and improved efficacy *in vivo*. | Fang et al., 2024 |
| Confocal Microscopy | ~ 150 nm MSNs - iRGD and PEOz | MDA-MB-231 spheroids | Dual-modified MSNs (PEOz and iRGD) enabled deep, uniform DOX distribution in MDA-MB-231 spheroids. Penetration was driven by iRGD-mediated targeting and trans-tissue transport, highlighting the carrier’s potential for improved solid tumour drug delivery. | Wang et al., 2024 |
| Confocal Microscopy | ~ 15 - 50 nm Au nanospheres and Au nanorods | A549 spheroids | Spherical AuNPs (10 - 50 nm) showed greater uptake and deeper spheroid penetration than rod-shaped AuNPs, likely due to more efficient membrane wrapping and easier navigation through the dense ECM. Rod-shaped NPs faced greater steric hindrance, limiting diffusion. Penetration of spherical NPs was dose-dependent, with AuNS50 occasionally reaching the spheroid core at higher concentrations. | Cybulski et al., 2025 |
| Confocal Microscopy | ~ 300 - 1000 nm polymeric particles with variations in size and charge | 4T1 and L929 spheroids | In cancer (4T1) spheroids, NP accumulation in proliferative, static, and necrotic zones was primarily influenced by particle size and charge. In normal (L929) spheroids, size and stiffness were the dominant factors. Imaging and flow cytometry enabled zone-specific mapping of NP distribution. | Parakhonskiy et al., 2025 |
| High-Content Screening Microscopy | 40 nm carboxylated polystyrene NPs | HT-29 spheroids | Confocal high-content screening in HT-29 spheroids showed that polystyrene NP penetration relied on active transport pathways regulated by Rab GTPases, highlighting molecular control of NP trafficking in 3D tumour models. | Cutrona and Simpson, 2019 |
| High-Content Screening Microscopy | 100 nm amine-modified NPs  100 nm carboxylated NPs | HepG2 spheroids | High-content imaging of HepG2 spheroids revealed variable cell responses to carboxylate- and amine-modified polystyrene NPs. Automated analysis and subcellular toxicity profiling underscored the importance of both large-scale and single-cell resolution in NP safety assessments. | Kelly et al., 2021 |
| Light-Sheet Fluorescence Microscopy and FRET | ~ 100 nm polyion complex micelles | MCF-7 MCTS | Simple PIC micelles rapidly released cargo after internalization in spheroid models. Light-sheet microscopy and FRET showed that introducing hydrophobic groups into the charged block improved micelle stability and controlled intracellular disassembly. | Chen et al., 2019 |
| Light-Sheet Fluorescence Microscopy | ~ 100 nm Doxorubicin-loaded polymer NPs | Panc-1/MRC-5/HUVEC hetero-spheroids | CLSM failed to capture full doxorubicin diffusion due to limited depth, while LSFM with image fusion revealed deep penetration into spheroid cores (up to 1 mm). Doxorubicin-loaded NPs were undetectable, emphasising delivery barriers in 3D models. | Lazzari et al., 2019 |
| Light-Sheet Fluorescence Microscopy | 70 nm and 120 nm fluorescent nanodiamonds and 20 nm and 100 nm polystyrene NPs | U87 spheroids | High-density nanodiamonds sedimented rapidly, reducing interaction with spheroids and resulting in limited, size-independent uptake. Extended exposure increased cell uptake. Nanodiamonds enabled biocompatible, long-term spheroid labelling and spatial cell isolation. | Niora et al., 2024 |
| Confocal and Light-Sheet Fluorescence Microscopy | ~ 10 - 100 nm Liposomes, PEGylated liposomes, lipoplexes, and reconstituted high-density lipoproteins (rHDL) | U87-MG spheroids | PEGylation had minimal impact on liposome penetration. Lipoplexes remained at the spheroid periphery due to positive charge and membrane fusion, while rHDL nanoparticles showed deep penetration and high accumulation within spheroids. | Niora et al., 2020 |
| Two photon FLIM and Confocal Microscopy | ~ 50 - 100 nm multimodal infrared probes | Live spheroid models based on HCT116, DPSC, and SKOV3 cells | Spheroid oxygenation was influenced by medium viscosity, size, and formation method. Some HCT116 and dental pulp spheroids showed “inverted” gradients, with higher core oxygen than the periphery. Microscopy revealed metabolic stratification, with glycolytic cores and oxidative cells at the edges. | Debruyne et al., 2024 |
| Two photon and synchrotron X-ray fluorescence microscopy | ~ 15 - 60 nm quasi-spherical AuNPs | MDA-MB-231 tumour spheroid model | In MDA-MB-231 spheroids, 15 nm NPs used an energy-independent transcellular route, 60 nm NPs used an energy-dependent pathway, and 22 nm NPs combined transcellular and paracellular transport, achieving the highest penetration efficiency. | Chen et al., 2024 |

# References

BROMMA, K., BECKHAM, W. & CHITHRANI, D. B. 2023. Utilizing two-dimensional monolayer and three-dimensional spheroids to enhance radiotherapeutic potential by combining gold nanoparticles and docetaxel. *Cancer Nanotechnology,* 14, 80. doi: 10.1186/Q12 s12645-023-00231-5

CHEN, F., RAVEENDRAN, R., CAO, C., CHAPMAN, R. & STENZEL, M. H. 2019. Correlation between polymer architecture and polyion complex micelle stability with proteins in spheroid cancer models as seen by light-sheet microscopy. *Polymer Chemistry,* 10, 1221-1230. doi: 10.1039/c8py01565a

CYBULSKI, P., BRAVO, M., CHEN, J. J.-K., VAN ZUNDERT, I., KRZYZOWSKA, S., TAEMAITREE, F., UJI-I, H., HOFKENS, J., ROCHA, S. & FORTUNI, B. 2025. Nanoparticle accumulation and penetration in 3D tumor models: the effect of size, shape, and surface charge. *Frontiers in Cell and Developmental Biology*, 12, 1520078. doi:10.3389/fcell.2024.1520078

DEBRUYNE, A. C., OKKELMAN, I. A., HEYMANS, N., PINHEIRO, C., HENDRIX, A., NOBIS, M., BORISOV, S. M. & DMITRIEV, R. I. 2024. Live microscopy of multicellular spheroids with the multimodal near-infrared nanoparticles reveals differences in oxygenation gradients. *ACS Nano,* 18, 12168-12186. doi: 10.1021/acsnano.3c12539

ELJE, E., MARIUSSEN, E., MORIONES, O. H., BASTÚS, N. G., PUNTES, V., KOHL, Y., DUSINSKA, M. & RUNDÉN-PRAN, E. 2020. Hepato (geno) toxicity assessment of nanoparticles in a HepG2 liver spheroid model. *Nanomaterials,* 10, 545. doi: 10.3390/nano10030545

FLEDDERMANN, J., SUSEWIND, J., PEUSCHEL, H., KOCH, M., TAVERNARO, I. & KRAEGELOH, A. 2019. Distribution of SiO2 nanoparticles in 3D liver microtissues. *International Journal of Nanomedicine*, 14, 1411-1431. doi: 10.2147/IJN.S189888

KELLY, S., BYRNE, M. H., QUINN, S. J. & SIMPSON, J. C. 2021. Multiparametric nanoparticle-induced toxicity readouts with single cell resolution in HepG2 multicellular tumour spheroids. *Nanoscale,* 13, 17615-17628. doi: 10.1039/d1nr04460e

MIKHAIL, A. S., EETEZADI, S. & ALLEN, C. 2013. Multicellular tumor spheroids for evaluation of cytotoxicity and tumor growth inhibitory effects of nanomedicines in vitro: a comparison of docetaxel-loaded block copolymer micelles and Taxotere. *PloS One,* 8, e62630. doi: 10.1371/journal.pone.0062630

NIORA, M., DUFVA, M., JAUFFRED, L. & BERG-SØRENSEN, K. 2024. Tumor Spheroid Uptake of Fluorescent Nanodiamonds Is Limited by Mass Density: A 4D Light-Sheet Assay. *Chemical & Biomedical Imaging*. 2025, 3, 6, 359-368. doi: 10.1021/cbmi.4c00088

PARAKHONSKIY, B., NOVOSELOVA, M., GORIN, D. & ABALYMOV, A. 2025. Comprehensive analysis of micro-and nanoparticle internalization in three-dimensional multicellular spheroids. *Applied Materials Today,* 42, 102534. doi: 10.1016/j.apmt.2024.102534

POPESCU, R. C., KOPATZ, V., ANDRONESCU, E., SAVU, D. I. & DOERR, W. 2023. Nanoparticle-mediated drug delivery of doxorubicin induces a differentiated clonogenic inactivation in 3D tumor spheroids in vitro. *International Journal of Molecular Sciences,* 24, 2198. doi: 10.3390/ijms24032198

ZHUANG, J., ZHANG, J., WU, M. & ZHANG, Y. 2019. A dynamic 3D tumor spheroid chip enables more accurate nanomedicine uptake evaluation. *Advanced Science,* 6, 1901462. doi: 10.1002/advs.201901462
